# p53 deficiency linked to B cell translocation gene 2 (BTG2) loss enhances metastatic potential by promoting tumor growth in primary and metastatic sites in patient-derived xenograft (PDX) models of triple-negative breast cancer

**DOI:** 10.1186/s13058-016-0673-9

**Published:** 2016-01-27

**Authors:** Emily Powell, Jiansu Shao, Yuan Yuan, Hsiang-Chun Chen, Shirong Cai, Gloria V. Echeverria, Nipun Mistry, Keith F. Decker, Christopher Schlosberg, Kim-Anh Do, John R. Edwards, Han Liang, David Piwnica-Worms, Helen Piwnica-Worms

**Affiliations:** Department of Cancer Biology, The University of Texas MD Anderson Cancer Center, Houston, TX 77030 USA; Department of Cancer Systems Imaging, The University of Texas MD Anderson Cancer Center, Houston, TX 77030 USA; Department of Biostatistics, The University of Texas MD Anderson Cancer Center, Houston, TX 77030 USA; Department of Bioinformatics and Computational Biology, The University of Texas MD Anderson Cancer Center, Houston, TX 77030 USA; Center for Pharmacogenomics, Department of Medicine, Washington University School of Medicine, St. Louis, MO 63110 USA

**Keywords:** Triple-negative breast cancer, Metastasis, p53, BTG2, PDX models

## Abstract

**Background:**

Despite advances in early diagnosis and treatment of cancer patients, metastasis remains the major cause of mortality. *TP53* is one of the most frequently mutated genes in human cancer, and these alterations can occur during the early stages of oncogenesis or as later events as tumors progress to more aggressive forms. Previous studies have suggested that p53 plays a role in cellular pathways that govern metastasis. To investigate how p53 deficiency contributes to late-stage tumor growth and metastasis, we developed paired isogenic patient-derived xenograft (PDX) models of triple-negative breast cancer (TNBC) differing only in p53 status for longitudinal analysis.

**Methods:**

Patient-derived isogenic human tumor lines differing only in p53 status were implanted into mouse mammary glands. Tumor growth and metastasis were monitored with bioluminescence imaging, and circulating tumor cells (CTCs) were quantified by flow cytometry. RNA-Seq was performed on p53-deficient and p53 wild-type tumors, and functional validation of a lead candidate gene was performed in vivo.

**Results:**

Isogenic p53 wild-type and p53-deficient tumors metastasized out of mammary glands and colonized distant sites with similar frequency. However, p53-deficient tumors metastasized earlier than p53 wild-type tumors and grew faster in both primary and metastatic sites as a result of increased proliferation and decreased apoptosis. In addition, greater numbers of CTCs were detected in the blood of mice engrafted with p53-deficient tumors. However, when normalized to tumor mass, the number of CTCs isolated from mice bearing parental and p53-deficient tumors was not significantly different. Gene expression profiling followed by functional validation identified B cell translocation gene 2 (*BTG2*), a downstream effector of p53, as a negative regulator of tumor growth both at primary and metastatic sites. BTG2 expression status correlated with survival of TNBC patients.

**Conclusions:**

Using paired isogenic PDX-derived metastatic TNBC cells, loss of p53 promoted tumor growth and consequently increased tumor cell shedding into the blood, thus enhancing metastasis. Loss of BTG2 expression in p53-deficient tumors contributed to this metastatic potential by enhancing tumor growth in primary and metastatic sites. Furthermore, clinical data support conclusions generated from PDX models and indicate that BTG2 expression is a candidate prognostic biomarker for TNBC.

**Electronic supplementary material:**

The online version of this article (doi:10.1186/s13058-016-0673-9) contains supplementary material, which is available to authorized users.

## Background

Metastases are responsible for the majority of deaths due to breast cancer. Metastasis is a multistage process that occurs through a sequence of steps involving dissociation of cells from the primary tumor, local invasion and migration through the basement membrane and into the circulation, extravasation into foreign organs, and finally tumor growth at distant sites [1]. Triple-negative (negative for estrogen receptor, progesterone receptor, and human epithelial receptor 2 (HER2) gene amplification) breast cancer (TNBC) is an aggressively metastatic subtype with a disproportionately high rate of *TP53* mutation compared to other breast cancer subtypes.

The tumor suppressor protein p53 is lost or mutated in about half of all human cancers, and in tumors where this gene (*TP53*) is wild-type (WT), mechanisms frequently exist to inactivate the protein. The majority of *TP53* mutations in basal-like breast cancer, an intrinsic breast cancer subtype that largely overlaps with TNBC, are insertions and deletions that result in *TP53* truncation and loss of function [2]. p53 loss disrupts pathways that inhibit metastasis and activates pathways that promote metastasis. The pathways that are altered by p53 loss regulate multiple stages of the metastatic cascade, including the acquisition of stem cell-like properties, interactions with the extracellular matrix, adhesion and migration [3, 4]. In addition, p53 loss disrupts cell cycle checkpoints and protects incipient tumor cells from undergoing apoptosis or entering senescence, which in turn, creates opportunities for tumor evolution and metastatic progression [5–7]. Furthermore, some *TP53* mutants confer additional functions that promote metastasis [8, 9]. Thus, the metastatic potential of tumors can be enhanced by loss of p53 or by expression of gain-of-function p53 mutants. However, studies conducted in vivo indicate that p53 loss alone is insufficient for metastasis [4, 8–10]. Interestingly, a genomic study of treatment-naïve TNBC revealed that p53 loss or acquisition of somatic mutations does not always emerge as a founding event [11], suggesting that disruption of p53 function also can influence late stages of tumor development. The presence of gain-of-function and loss-of-function mutations in breast cancer warrants a thorough characterization of these mutations in tumor progression. In this study, we specifically studied the contribution made by p53 deficiency to metastasis in late-stage triple-negative breast cancer.

Most of the existing preclinical breast cancer xenograft models used to study metastasis to lung or bone involve injecting human cancer cell lines that have been extensively cultured ex vivo into the tail vein or left ventricular chamber of the heart, respectively. These methodologies bypass all early steps in the metastatic cascade, including escape from the primary site and survival in circulation. By contrast, orthotopic patient-derived xenograft (PDX) models of breast cancer are derived by engrafting tumors obtained directly from patients into the mammary fat pads of immune-compromised mice. Human breast tumors have been shown to metastasize to physiologically relevant organs in these models, and as such, orthotopic PDX models enable all stages of the metastatic cascade to be studied within a more advanced biological context in vivo [12–15].

We engineered paired isogenic PDX lines differing only in p53 status to develop a breast cancer metastasis model of TNBC that enabled longitudinal studies in mice [16]. By studying the effect of p53 loss in an already-metastatic PDX line, we investigated whether p53 loss impacted late stages of tumor progression by examining various stages of the metastatic cascade over time. The contributions made by p53 silencing to breast tumor growth, escape from the mammary gland, homing and colonization of distant organs, and tumor growth at metastatic sites were investigated. In addition, gene expression profiling was conducted to identify p53 effectors that regulate metastasis.

## Methods

### Study approval

This study was carried out in strict accordance with the recommendations in the Guide for the Care and Use of Laboratory Animals from the National Institutes of Health (NIH) Institutional Animal Care and Use Committee (IACUC). The protocol was approved by the Committee on the Ethics of Animal Experiments of Washington University and the IACUC at MD Anderson Cancer Center. Mice were euthanized when they became moribund and when they reached defined study end points. Animals were euthanized as dictated by the Association for Assessment and Accreditation of Laboratory Animal Care International and IACUC euthanasia end points.

### Establishment of PDX models of TNBC

PDX models were established according to published protocols [17]. Briefly, 2.5 × 10^5^ immortalized human mammary stromal fibroblasts, derived from a patient who underwent a reduction mammoplasty, were irradiated (400 Rads) and mixed with 2.5 × 10^5^ nonirradiated cells. Fibroblasts were then mixed with 1 × 10^6^ tumor cells stably expressing click beetle red luciferase (CBR-luc)/mCherry and 1/3 volume Matrigel. This mixture was injected into the fourth mammary fat pads of 3- to 4-week-old nonobese diabetic/severe combined immunodeficiency (NOD/SCID) mice (ordered from the National Cancer Institute). For metastasis studies, p53-deficient tumors were resected before they reached 1 cm in diameter (approximately 9 weeks post-engraftment) to enable time for metastasis to occur before mammary tumor burden reached the maximum allowable size. For studies monitoring mammary tumor growth, tumors were not resected and mice were euthanized when tumors reached 2 cm in diameter.

### Bioluminescence imaging

Bioluminescence imaging (BLI) was performed in vivo as previously described [18]. Briefly, after injection of 150 μg/g D-luciferin (Biosynth, Staad, Switzerland) in phosphate-buffered saline (PBS), intraperitoneal (i.p.), isoflurane-anesthetized mice were imaged with a charge-coupled device camera-based BLI system (IVIS Lumina and IVIS Spectrum; PerkinElmer, Waltham, MA, USA). Signals were displayed as photons/s/cm^2^/sr. Regions of interest were defined manually using Living Image Software, and data were expressed as total photon flux (photons/second). The first images were taken 2–3 weeks after tumor implantation (PDX models), or immediately after tail vein injection of labeled cells, and weekly thereafter. To assess the appearance of metastases in the axillary lymph node, mammary tumors were covered to block signal from the primary site and allow visualization of metastases. To quantify organ distribution, D-luciferin was administered to live animals, and tissues were assessed with BLI ex vivo at necropsy.

### Quantification of metastatic frequency and manual counting of metastatic foci

Mice were euthanized a minimum of 19 weeks post-engraftment, and organs were subjected to BLI ex vivo. Organs exhibiting regions of bioluminescence with Gaussian distribution were counted as one nodule.

### Blood processing for circulating tumor cell (CTC) analysis

Blood was extracted by cardiac puncture in the presence of heparin. Whole blood was immediately transferred to red blood cell (RBC) lysis buffer (Sigma-Aldrich, St. Louis, MO, USA; R7757), inverted 10 times, and placed on ice. Samples were centrifuged at 1500 rpm for 6 min, RBC lysis buffer was carefully removed, and an additional 7.5 ml of RBC lysis buffer was added. Samples were again centrifuged at 1500 rpm for 6 min. Pellets were resuspended in 350 μl of PBS with 0.5 % bovine serum albumin (BSA) and assessed immediately by flow cytometry. Whole blood from non-tumor-bearing mice was processed alongside tumor-bearing mice as negative controls. Whole blood from non-tumor-bearing mice was spiked with 20,000 BC3-p53 knockdown (KD) cultured cells as a positive control.

### Flow cytometry for circulating tumor cells

For CTC studies, samples were analyzed on an Influx cell sorter (BD, Franklin Lakes, NJ, USA). The entire sample was flowed regardless of total cell number. Dead cells were gated out by SyTox Blue staining and forward/side scatter. Gates were set such that no cells appeared in the mCherry + gate in negative controls. CTCs were identified as cells positive for mCherry fluorescence.

### Cell culture and lentiviral transduction of Washington University Breast Cancer line 3 (WU-BC3) cells

BC3-p53WT and BC3-p53KD cells were grown in Dulbecco’s modified Eagle’s medium (DMEM) supplemented with 10 % fetal bovine serum (FBS) and 1 % penicillin-streptomycin. Cells were cultured at 37 °C, 5 % CO_2_, and 5 % O_2_ in humidified air. When logarithmically growing cells reached approximately 50 % confluency, they were transduced with lentivirus encoding CBR-luc and mCherry (FUW-CBR-luc-mCherry) in the presence of 1 μg/ml polybrene. Cells were selected and subsequently grown in the presence of puromycin. Lentivirus encoding BTG2 was produced by transfecting 293 T cells with the target plasmid along with the packaging vectors pDELTA8.9 and VSVG. Virus was removed from cells and passed through a 40 μm filter 72 h after transfection. To express BTG2 in BC3-p53KD, cells were transduced with pHAGE-BTG2 or control lentivirus (pHAGE-GFP-eGFP). Cells were transduced at a multiplicity of infection (MOI) of 1 in the presence of 10 μg/ml polybrene. Flow cytometry was used to isolate green fluorescent protein (GFP)-positive cells. Expression of BTG2 was assessed by quantitative polymerase chain reaction (qPCR).

### Ribonucleic acid-sequencing (RNA-Seq) experiments and data analysis

Tumors were harvested when they reached 0.5 cm and were immediately snap frozen in liquid nitrogen to preserve RNA integrity and to avoid experimental manipulation that could lead to alterations in gene expression. Tumors were stored at -80 °C, thawed on ice in Trizol, and homogenized with a pedestal homogenizer. Total RNA was extracted with chloroform followed by purification using the RNEASY kit (Qiagen, Venlo, The Netherlands; 74104). Total RNA was treated with DNase (TURBO DNase, Life Technologies, Carlsbad, CA, USA; AM2238) followed by column purification (RNA Clean & Concentrator-5, Zymo Research, Irvine, CA, USA; R1015). mRNA was isolated by poly-A selection. RNA Integrity Number (RIN) was assessed on a Bioanalyzer (Agilent Technologies, Santa Clara, CA, USA), and only samples with RIN ≥ 8 were sequenced. RNA-Seq libraries were prepared and sequencing was performed by the Genome Technology Access Center (Washington University, St. Louis, MO, USA). The RNA-Seq reads were first mapped to the mouse (NCBI Build 37.2) (GRCm37UCSC mm9) reference genome using Tophat2, allowing a maximum of two mismatches per 75 bp read end. The unmapped reads were then aligned to the human genome (NCBI Build 37.2) (GRCh37/UCSC hg19) using the same tool and parameters. Partek Genomics Suite v6.6 and its genomic feature database (RefSeq Transcripts - 2014-01-03) were used to quantify the gene-level read counts. The differential analyses for gene/isoform expression were performed using DESeq2. The log2 fold change 1.5 and *p* value cutoff <0.05 were used to identify differentially expressed genes. Pathway analysis was performed using the GeneGo application of the MetaCore program (Thomson Reuters, New York, NY, USA).

### Real-time polymerase chain reaction (RT-PCR)

Total RNA was isolated by Trizol-chloroform extraction followed by purification using the RNEASY kit as described above. RNA was DNase-treated as described above. RIN was assessed as described above, and only samples with RIN ≥8 were used for qPCR analyses. A dynamic range test (standard curve) was performed using reverse transcription (RT) conditions to determine the range of RNA to be used in all subsequent reactions. Each 40 μl RT reaction consisted of no more than 4 μg template RNA. RNA was diluted 1:10 and reverse transcribed using the Superscript III system (Invitrogen, Carlsbad, CA, USA). The resulting cDNA was used for qPCR using the TaqMan Gene Expression Assays (Applied Biosystems, Foster City, CA, USA), and data were normalized to a multiplexed endogenous control, GAPDH. No-template and no-RT controls were run on each plate, and amplification was not observed for any samples. qPCR was performed on the ViiA 7 Real-Time PCR System (Life Technologies).

### Immunohistochemistry (IHC)

Mammary tumors were fixed in 10 % neutral buffered formalin for 24–48 h. Samples were then washed three times with PBS and transferred to 70 % ethanol, then embedded in paraffin. Five micron sections were cut and baked at 65 °C for 60 min. Sections were deparaffinized by immersing in xylene (Thermo Fisher Scientific, Waltham, MA, USA) three times for 5 min each and rehydrated by immersing twice through a decreasing gradient of alcohol (100 %, 95 %, 70 %, 50 %, and distilled H_2_0) for 2 min each. Antigen retrieval was carried out by boiling samples in rodent decloaker agent (Biocare Medical, Concord, CA, USA; RD913M) for 30 min followed by cooling at room temperature for an additional 15 min. Endogenous peroxidase activity was blocked by incubating sections in Peroxidase Blocking Reagent (Dako, Glostrup, Denmark) for 15 min at room temperature. Nonspecific interactions were blocked by incubating sections in Protein Block (Dako) for 1 h at 4 °C. Primary and secondary antibodies were diluted in Antibody Diluent (Dako). Antibodies were diluted as follows: cytokeratin 18 (CK-18) (Abcam, Cambridge, UK; Ab82254), 1:200; phospho-histone H3 (pHH3) (Sigma-Aldrich; H9908), 1:1500; cleaved caspase 3 (CC3) (Cell Signaling Technology, Danvers, MA, USA; 9661), 1:200. For CC3, the ImmPRESS Reagent Anti-Rabbit IgG kit was used (Vector Laboratories, Burlingame, CA, USA; MP-7401). For pHH3, the ImmPRESS Reagent Anti-Rat IgG kit was used (Vector Laboratories MP-7404). For CK-18, the ImmPRESS M.O.M kit (Vector Laboratories; MP-2400) was used.

### Statistics

To normalize the photon flux values to the time of study end point, the photon flux and the logarithm of the photon flux in each organ were plotted versus time to euthanasia. Plots of the photon flux showed that most values increased approximately exponentially over time, and plots of the logarithm of the photon flux indicated that most values were fitted by a straight line [19, 20]. We therefore assumed that the photon flux increased exponentially over time. Thus, the normalized photon flux was obtained by dividing the photon flux measured in each by the exponential value of its time to euthanasia. Since the sample sizes in most groups were relatively small, Wilcoxon rank sum test was used to evaluate the mean differences in the photon flux values in each organ, time to euthanasia, and time to lymph node metastasis between the groups of mice. F-test was used to account for the time effect in the analysis of variance (ANOVA) model to assess whether p53 silencing was significantly correlated with the log2-transformed CTC counts. Statistical analyses were performed using R (http://www.r-project.org/).

### Survival analysis

The univariate Cox proportional hazard model was used to assess the correlation of gene expression with patient overall survival and metastasis-free survival (Table S3 in Additional file 1), and the likelihood ratio test *p* values were reported. The dataset from The Cancer Genome Atlas (TCGA) [21] was used for determining overall survival of breast cancer patients and TNBC patients. Datasets from the San Diego cohort [22], the NKI cohort [23], and the Oxford cohort [24] were used for determining metastasis-free survival. Log-rank tests were used to estimate the *p* values between high and low expression groups (using top and bottom 25 % as the cutoff for grouping).

### Preparation of tumors for flow cytometery for stem markers and mammosphere formation assays

For use in flow cytometry and mammosphere assays, BC3-p53WT and BC3-p53KD tumor cells not expressing CBR-luc-mCherry were implanted contralaterally into the fourth mammary gland (n = 4 tumors BC3-p53WT, n = 4 tumors BC3-p53KD). When tumors reached 0.5 cm in diameter, they were isolated from mammary glands and dissociated into single cells and organoids in collagenase and hylauronidase for 4 h at 37 °C on a rotator. Tumor cells were washed in DMEM/F12 with 5 % bovine calf serum, and red blood cells were lysed for 5 min at room temperature (RBC lysis buffer; Sigma-Aldrich). Cells were passed through a 100 μm, then 40 μm filter. Mouse stromal cells were depleted using magnetic-activated cell sorting (Miltenyi Biotec, Bergisch Gladbach, Germany; MACS mouse cell depletion resin) according to the manufacturer’s instructions. Effective depletion of mouse cells was confirmed by staining with a mouse-anti-H2kd (mouse-specific MHC class I H-2K^d^ haplotype) antibody conjugated to PE/Cy7 (BioLegend, San Diego, CA, USA clone SF1-1.1), followed by flow cytometry.

### Flow cytometery

Following tumor digestion and mouse cell depletion, cells were immediately placed in PBS + 0.5 % BSA and stained with antibodies for flow cytometry for 20 min at 4 °C at a concentration of 5 million cells/mL. Antibodies used were: rat anti-CD44 conjugated to allophycocyanin (APC) (BD Pharmingen San Jose, CA, USA; clone IM7), mouse anti-CD24 conjugated to fluorescein isothiocyanate (FITC) (BD Pharmingen clone ML5), mouse anti-GD2 (unconjugated, BD Pharmingen clone 14.G2a), goat-anti-mouse secondary antibody conjugated to APC (BD Pharmingen). Cells were stained with Ghost Dye Violet 510 (Tonbo Biosciences, San Diego, CA, USA) for viability. After staining, cells were fixed in 1 % paraformaldehyde then analyzed by an LSRFortessa X-20 flow cytometer (BD).

### Mammosphere formation assays

Immediately following tumor digestion and mouse cell depletion, cells were plated at a concentration of 1000 cells per well on an ultra-low-binding 96-well plate (Corning, New York, NY, USA) in complete MammoCult medium (STEMCELL Technologies, Vancouver, BC, Canada), supplemented with 1 % methylcellulose to immobilize cells. Mammospheres were manually counted 12 days after plating. Primary mammosphere formation efficiency was calculated as percent of mammospheres formed divided by total number of cells plated. Primary mammospheres were collected, dissociated into single cells using TrypLE Express (Life Technologies), and replated in MammoCult containing 1 % methylcellulose to form secondary mammospheres. Secondary mammospheres were manually counted 12 days after plating.

### 3-(4,5-dimethylthiazol-2-yl)-2,5-diphenyltetrazolium bromide (MTT) assays

Cells were seeded in 24-well plates (20,000 cells per well) in complete cell culture media. Four consecutive time points were harvested post-seeding (24, 48, 72 and 96 h). Every 24 h, plates were incubated with MTT reagent (0.5 mg/ml) for 1 h at 37 degrees, 5 % CO2, and 5 % O2 in humidified air (normal cell culture conditions). After incubation with MTT reagent, media was removed from cells, and 500 μl DMSO was added per well to solubilize purple formazan crystals. The plate was then read on a Clariostar plate reader (BMG Labtech, Cary, NC, USA) at 570 nm for formazan absorbance and 690 nm for background absorbance, and these values were normalized by subtraction. Each condition was performed in triplicate.

### Cell proliferation assays

Cells were seeded in 6-well plates (80,000 cells per well) in complete cell culture media. Four consecutive time points were harvested post-seeding (24, 48, 72 and 96 h). Every 24 h, cells were trypsinized, resuspended to break remaining cell clusters, loaded onto cell counting chambers, and read on a CellOMeter (Nexcelom Bioscience, Lawrence, MA, USA). Each condition was plated in biological triplicates, and each well was counted in duplicate. Each chamber was read in triplicate.

### Western blotting

Samples were lysed in Mammalian Cell Lysis Buffer (50 mM Tris, pH 8.0, 2 mM dithiothreitol (DTT), 5 mM ethylenediaminetetraacetic acid (EDTA), 0.5 % NP-40, 100 mM NaCl) with added protease inhibitors (1 μM mycrocystin, 2 mM phenylmethanesulfonyl fluoride (PMSF), 1 × protease inhibitor cocktail (Sigma-Aldrich; P8340-5ML), 1 × phosphatase inhibitor cocktail (Calbiochem, San Diego, CA, USA; 524625-1SET)), and 10–30 μg total protein was loaded onto Bio-Rad (Hercules, CA USA) Criterion gels and transferred to nitrocellulose for Western blotting using standard procedures. Antibodies used for Western blotting included those recognizing BTG2 (sc-33775, Santa Cruz Biotechnology Inc., Dallas, TX, USA), and α-tubulin (2144, Cell Signaling Technologies). Protein was detected using enhanced chemiluminesence (ECL) (34080, Thermo Fisher Scientific) on a G-Box imager (Syngene, Frederick, MD, USA).

### Ethics, consent, and permission

All animal experiments were authorized by the IACUC committee at MD Anderson. RNA-seq data analyzed in this manuscript are publically available at (GEO Accession number GSE76433). We confirm that this study did not involve human patients and no consent was required.

## Results

### Modeling human breast cancer metastasis in mice

The original line (WU-BC3) was previously generated by engrafting the primary breast tumor of a patient with metastatic TNBC directly into the humanized mammary fat pad of NOD/SCID mice [16]. This patient presented with metastases to lung, bone, adrenal gland, and mediastinal lymph nodes. DNA sequencing revealed that this tumor is wild-type (WT) for *TP53* [25], and furthermore, p53 is known to be functional in these cells (both p53 and p21 were induced in response to ionizing radiation) [16]. Gene expression profiling and application of the PAM50 subtype-based predictor categorized WU-BC3 as nonbasal TNBC that clustered most closely with the HER2-E subtype [26]. The HER2-E molecular subtype has been reported in approximately 9 % of TNBC and approximately 30 % of HER2-E breast cancers are WT for *TP53* [21]. WU-BC3 cells were infected with control retroviruses or retroviruses encoding p53-specific short hairpin RNAs (shRNAs) [16] to generate cells denoted BC3-p53WT and BC3-p53KD, respectively, which were then further engineered to stably express both bioluminescent (click beetle red luciferase, CBR-luc) and fluorescent (mCherry) markers by lentiviral transduction (Figure S1A in Additional file 2). Silencing of p53 in WU-BC3 caused an increase in cellular proliferation in vitro (Figure S1B and C in Additional file 2). The morphology of the BC3 line was not detectably altered (Figure S1D in Additional file 2). Transduced cells were implanted into the humanized mammary fat pads of NOD/SCID mice. Thus, isogenically matched PDX lines differing only in p53 status were used throughout our studies, enabling tumor growth and metastasis to be monitored noninvasively and repetitively in living mice and in organs isolated from these mice using bioluminescence imaging (BLI).

### p53 deficiency results in increased tumor growth in mammary glands

**Fig. 1:**
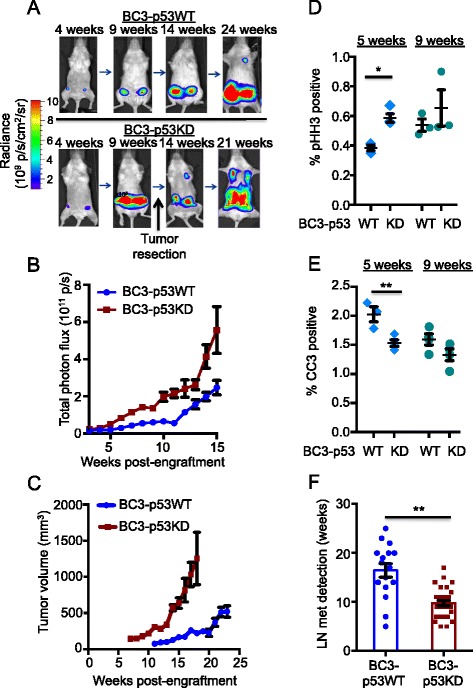
p53 silencing enhances breast tumor growth and metastasis in vivo. BC3-p53WT and BC3-p53KD cells expressing CBR-luc and mCherry were implanted into the humanized mammary fat pads of NOD/SCID mice. Bioluminescence imaging (BLI) was performed to monitor tumor growth and metastasis as a function of time. **a** Representative images showing growth and metastasis of BC3-p53WT and BC3-p53KD tumors. Due to rapid tumor growth BC3-53KD tumors were resected at approximately 9 weeks post-engraftment. **b** Total photon flux from each mammary tumor was quantified from BLI, and values were plotted as a function of time. **c** Tumors were measured with calipers, and values were plotted as a function of time. n = 18, BC3-p53WT; n = 18, BC3-p53KD. *p* = 0.004, *t* test (**b** and **c**). **d** and **e** 5 weeks and 9 weeks post-engraftment, tumors were harvested, formalin-fixed, sectioned, and stained with antibodies against phospho-histone H3 (pHH3) (**d**) and cleaved caspase 3 (CC3) (**e**). Positive regions were counted manually, and percentage of positive cells was calculated from DAPI-stained nuclei. Each data point is the average of at least six images from an individual mouse. Paired *t* tests, *p* = 0.01 for pHH3 at 5 weeks; *p* = 0.30 for pHH3 at 9 weeks; *p* = 0.04 for CC3 at 5 weeks; *p* = 0.20 for CC3 at 9 weeks. **f** BLI was performed biweekly to detect the appearance of lymph node metastases. *p* = 0.001, Wilcoxon rank sum test. Each data point represents one mouse. All error bars represent standard error of the mean (SEM)

The paired BC3-p53WT and BC3-p53KD cells were engrafted into mammary fat pads of individual NOD/SCID mice and BLI was performed to follow tumor growth and metastasis as a function of time (Fig. 1a). BLI and caliper measurements revealed that p53-deficient tumors grew faster than their p53 WT counterparts (Fig. 1b, c). Immunohistochemistry was used to determine if the increased growth of p53-deficient tumors in the mammary gland was due to greater proliferation and/or less apoptosis. Five weeks after tumor implantation, p53-deficient tumors exhibited a significant increase in proliferating cells (Fig. 1d) and a significant decrease in apoptotic cells (Fig. 1e). By 9 weeks post-engraftment, proliferation and apoptosis were not significantly different, which was consistent with the observation that tumor growth rates equalized at later time points (Fig. 1b,c). In addition to faster primary tumor growth, metastasis to the axillary lymph node was observed earlier in mice harboring p53-deficient tumors (Fig. 1f). This suggested that BC3-p53KD tumors completed selected stages of the metastatic cascade more efficiently than BC-p53WT tumors.

### p53 deficiency leads to increased growth at metastatic sites

**Fig. 2:**
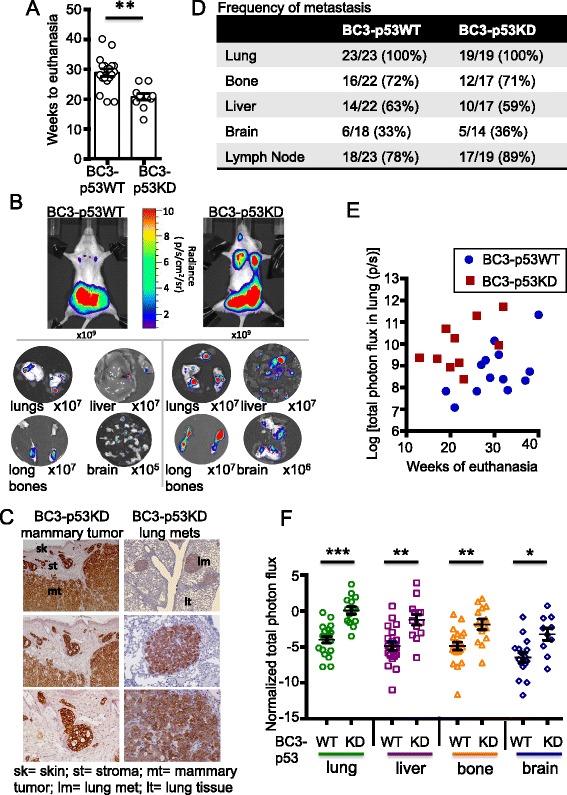
p53 silencing increases bioluminescence in metastatic sites. BC3-p53WT and BC3-p53KD were implanted into mouse mammary fat pads. Frequency and magnitude of metastasis were assessed with bioluminescence imaging (BLI). **a** Animals were euthanized when declining health was observed. The time post tumor-engraftment to euthanasia of animals implanted with BC3-p53WT or BC3-p53KD was quantified. Each data point represents one mouse. *p* = 0.002, Wilcoxon rank sum test. **b** Lungs, livers, long bones, and brains of mice implanted with BC3-p53WT or BC3-p53KD were imaged with BLI ex vivo at necropsy. Representative images are shown. Scale applies to each image in the panel, and magnitude of scale is indicated below each image. **c** Mammary tumors and lungs were harvested from mice implanted with BC3-p53KD. Tissues were sectioned and stained with cytokeratin 18 (CK18) to assess regions of human epithelial tumor (positive) and surrounding mouse tissue (negative). Objectives used were: 20 × composite (*top panels*), 10 × (*middle panels*), and 20 × (*bottom panels*). **d** Frequency of metastasis to the indicated organs was quantified with ex vivo BLI 19–40 weeks post-engraftment. **e** Lungs were extracted, and total photon flux was assessed with BLI, quantified, and plotted vs. time post-engraftment to study end point. *p* = 0.03, linear regression analysis of the slopes. **f** Lungs, livers, bones, and brains were extracted and assessed with BLI. Total photon flux was quantified and normalized to time post tumor-engraftment to euthanasia. n = 21, p53WT; n = 14, p53KD. *p* <0.001 (lung); *p* = 0.001 (liver); *p* = 0.002 (bone); *p* = 0.01 (brain), Wilcoxon rank sum tests. Each data point represents one organ from one mouse. All error bars represent standard error of the mean (SEM)

Tumor-bearing mice were monitored for signs of declining health and euthanized when they exhibited moribund symptoms, typically at approximately 30 and approximately 20 weeks post-engraftment of BC3-p53WT and BC3-p53KD tumors, respectively (Fig. 2a). At the time of euthanasia, organs were isolated and subjected to BLI ex vivo. Metastases were observed in lungs, livers, bones, and brains of tumor-bearing mice (Fig. 2b). Immunohistochemistry using a human-specific cytokeratin 18 (CK18) antibody confirmed that primary and metastatic lesions were of human origin (Fig. 2c). p53 silencing did not alter the frequency of metastasis to various organs (Fig. 2d). However, even though mice bearing BC3-p53KD tumors were euthanized earlier than mice bearing BC3-p53WT tumors (Fig. 2a), the photon flux measured in the lungs of mice harboring p53-deficient tumors was higher than that measured in the lungs of mice harboring p53WT tumors at the study end points (Fig. 2e). Bioluminescence of lungs, livers, bones, and brains was significantly greater in mice bearing p53-deficient tumors, even when photon flux was normalized to the date of euthanasia (Fig. 2f). Normalization to photon flux of mammary tumors was not possible because they were resected in a survival surgery prior to the study end point. This suggested that p53 silencing enhanced the metastatic capacity of the BC3 tumor line.

Limiting dilution cell transplantation assays were performed to determine if the number of tumor-initiating cells increased upon p53 silencing. The results of this assay demonstrated that the majority of both BC3-p53WT and BC3-p53KD cells were capable of tumor initiation (Figure S2A, B in Additional file 3). Tumors were also dissociated into single cell suspensions and assessed for their ability to grow as nonadherent mammospheres, a property of tumor-initiating cells [27]. The ability of BC3 tumor cells to initiate primary (Figure S2C in Additional file 3) and secondary (Figure S2D in Additional file 3) sphere formation was not significantly changed upon p53 silencing. Finally, p53 silencing did not significantly alter the percentage of CD44^high^/CD24^low^ cells [28] in the tumor cell population (Figure S2E in Additional file 3). Taken together, these data demonstrated that p53 silencing did not significantly alter the stem-like properties of these breast cancer cells.

To determine the effect of p53 silencing on the ability of breast tumor cells to colonize and grow in distant organs, cells were injected to the tail vein (for lung metastases) or the left ventricular chamber of the heart (for bone metastases). Bioluminescence was tracked in vivo. Silencing of p53 resulted in faster tumor growth in the bones (Figure S3A in Additional file 4) and lungs (Figure S3B in Additional file 4) of injected mice. Thus, p53 silencing promoted tumor growth in both primary (mammary gland) and secondary (lung and bone) sites.

### Circulating tumor cell (CTC) release correlates with tumor growth

**Fig. 3:**
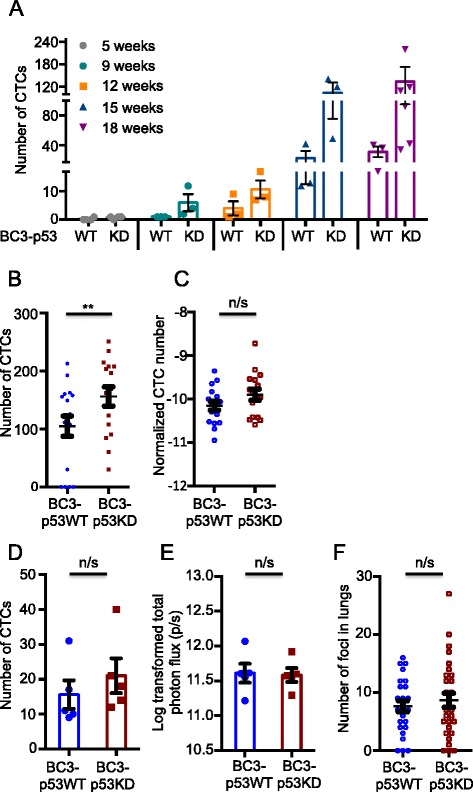
p53 silencing increases CTCs in the blood as a function of increased tumor growth. Mouse mammary fat pads were engrafted with BC3-p53WT or BC3-p53KD. Whole blood was extracted from mice in a terminal blood draw by cardiac puncture. Red blood cells were lysed, and circulating tumor cells (CTCs) were assessed by flow cytometry for mCherry-positive cells. Bioluminescence imaging (BLI) was performed on mammary tumors and lungs at necropsy. **a** CTCs were quantified by flow cytometry at the indicated time points. *p* = 0.71 (5 weeks); *p* = 0.06 (9 weeks); *p* = 0.40 (12 weeks); *p* = 0.10 (15 weeks); *p* = 0.07 (18 weeks), Wilcoxon rank sum tests. **b** CTCs were quantified as in (**a**), and numbers from each time point were combined to increase cohort size. *p* <0.001, F-test. **c** CTC number from each mouse was normalized to total photon flux of the corresponding primary tumor. Data are presented as a combination of all time points as in (**b**). *p* = 0.072, F-test. **d** and **e** BC3-p53WT tumors were implanted to mammary glands 3 weeks prior to implantation of BC3-p53KD, and CTCs were quantified on the same day by flow cytometry (**d**, *p* = 0.43, *t* test) when tumors were equivalent in size (**e**, *p* = 0.73, *t* test). **f** Spatially distinct regions of bioluminescence in lungs of tumor-bearing mice were quantified manually. *p* = 0.09, Wilcoxon rank sum test. Each data point represents one mouse. All error bars represent standard error of the mean (SEM)

To assess the ability of tumor cells to enter into the bloodstream, CTCs present in the blood of tumor-bearing animals were quantified by flow cytometry at various times following tumor engraftment. The number of CTCs able to metastasize out of the mammary glands of tumor-bearing mice increased as a function of time, and greater numbers of CTCs were detected in mice harboring BC3-p53KD tumors than those harboring BC3-p53WT tumors at each time point examined (Fig. 3a). CTCs were not detected in either line prior to 9 weeks post-implantation. The total number of CTCs released over the 18-week period by mice harboring BC3-p53KD tumors was significantly higher than the total number released by mice harboring BC3-p53WT tumors (Fig. 3b). However, when total CTC numbers were normalized to photon flux (Fig. 3c, Figure S4A in Additional file 5) or volume (Figure S4B in Additional file 5) of the corresponding mammary tumor, the difference was not statistically significant. p53 status did not influence CTC numbers (Fig. 3d) from mice bearing equal-sized tumors (Fig. 3e), validating this normalization. Similarly, CTC number correlated with intensity of photon flux (Figure S4C in Additional file 5) and volume (Figure S4D in Additional file 5) of mammary tumors within each mouse. The number of CTCs also correlated with photon flux in the lungs of tumor-bearing mice (Figure S4E in Additional file 5), suggesting that our method of CTC detection effectively measured CTCs that would eventually seed metastatic lesions. The number of spatially distinct metastatic lesions in the lungs of tumor-bearing mice was not significantly altered when p53 was silenced (Fig. 3f), suggesting that p53 deficiency did not influence the ability of CTCs to colonize the lungs. As expected, CTC number did not correlate with time of detection of lymph node metastasis (Figure S4F in Additional file 5). Taken together, these data indicated that p53 silencing did not increase the ability of tumor cells to escape the mammary glands, to home to the lung, or to colonize the lung. Rather, increased tumor growth in primary and metastatic sites was likely responsible for the observed increases in tumor progression and metastasis upon p53 silencing.

### p53 silencing alters expression of genes involved in tumor progression

RNA-sequencing (RNA-Seq) was performed on BC3-p53WT and BC3-p53KD mammary tumors to identify genes with altered expression in response to p53 silencing. Tumors were harvested when they reached approximately 0.5 cm in diameter. Using a bioinformatics pipeline that distinguishes human epithelial tumor cell transcripts from transcripts originating from the murine host (see Methods), we identified 109 significantly upregulated genes and 51 significantly downregulated genes in human tumor cells from BC3-p53KD tumors (n = 3) relative to BC3-p53WT tumors (n = 4) (using a cutoff value of *p* ≤0.05 and log2 fold change ≥1.5) (Table S1 in Additional file 6). p53 silencing resulted in a 10.9-fold reduction in *TP53* expression in BC3-p53KD tumors relative to BC3-p53WT tumors. The top enriched processes differentially affected by p53 silencing were those related to interactions with the surrounding microenvironment, including extracellular matrix (ECM) remodeling, connective tissue degradation, cell-matrix interactions, cell adhesion, epithelial to mesenchymal transitions (EMT) and apoptosis (Table S2 in Additional file 7). Alterations in components of these pathways are known to contribute to various stages of metastasis [29, 30], including growth in primary and metastatic sites [29, 30].

### Gene expression profiling identifies p53-regulated genes that are clinically significant

Changes in gene expression that accompany disruptions in the p53 pathway are associated with poor prognosis in breast cancer patients [31–37]. The gene expression signature derived in this study was reviewed to create a mini-signature of genes known to be in the p53 pathway. The dataset from The Cancer Genome Atlas (TCGA) [21] was then used to determine if the expression (or lack thereof) of each gene in the mini-signature was predictive of overall survival for breast cancer patients (n = 1022) as well as for those patients with TNBC (n = 119). Datasets from the San Diego cohort (n = 286) [22], the NKI cohort (n = 295) [23] and the Oxford cohort (n = 210) [24] were used to correlate gene expression with metastasis-free survival (Table S3 in Additional file 1). B cell translocation gene 2 (BTG2) was chosen from the mini-gene signature for validation and further analysis.

### BTG2 regulates growth of primary and metastatic tumors

**Fig. 4:**
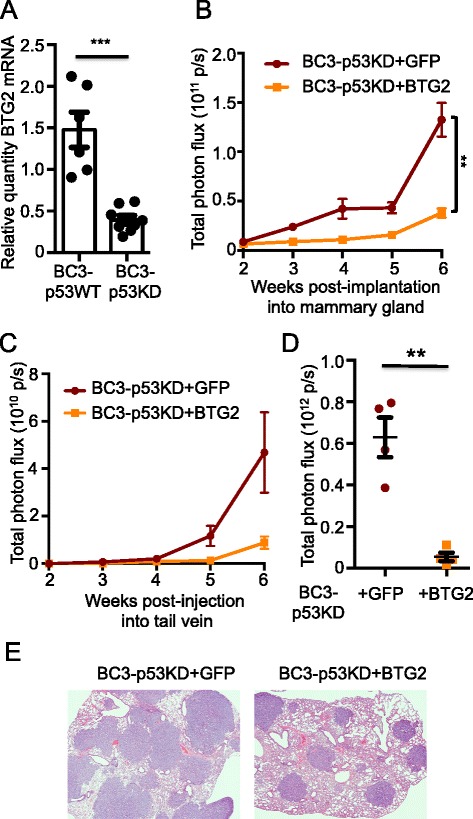
BTG2 negatively regulates growth of BC3-p53KD tumors in primary and metastatic sites. BC3-p53WT or BC3-p53KD were implanted into mouse mammary fat pads, and tumors were harvested when they reached 0.5 cm diameter. Ribonucleic acid (RNA) was extracted from tumors, and gene expression changes were assessed with RNA-sequencing (RNA-Seq). **a** RNA from BC3-p53WT or BC3-p53KD tumors was reverse transcribed, and quantitative real-time polymerase chain reaction (qRT-PCR) using a probe and primer set for B cell translocation gene 2 (BTG2) was performed. *p* = 0.0001, *t* test. Each dot is a biological replicate representing one tumor from one mouse. **b** BTG2 or green fluorescent protein (GFP) (control) was expressed in BC3-p53KD cells by lentiviral transduction, and cells were implanted to mouse mammary fat pads. Mice were subjected to bioluminescence imaging (BLI) weekly, and total photon flux from mammary tumors was quantified at each time point. n = 5 mice (10 tumors) in each group. *p* <0.001 for mammary tumors 6 weeks post-engraftment, Wilcoxon rank sum test. *p* <0.001 using linear mixed-effect model of tumor growth over the time course. **c** BTG2 or GFP was expressed in BC3-p53KD as in (**b**), and cells were injected into mouse tail veins to model the final stages of lung metastasis. Mice were subjected to BLI weekly, and total photon flux from lungs was quantified at each time point. n = 4 mice in each group. *p* = 0.002, linear mixed effect model. **d** Total photon flux from lungs of mice in (**c**) was assessed with BLI ex vivo at necropsy and quantified. *p* = 0.001, *t* test. Each data point represents one mouse, n = 4 mice per group. **e** Hematoxylin and eosin (H & E) staining of lungs of mice in (**c**) and (**d**). All error bars represent standard error of the mean (SEM)

BTG2 is a direct transcriptional target of p53, and quantitative RT-PCR (qPCR) validated that basal BTG2 expression was dependent on the p53 status of the originating cell lines (Fig. 4a) [38–40]. We then ectopically expressed BTG2 in BC3-p53KD cells, using GFP expression as a control (Figure S5A, B in Additional file 8). Ectopic expression of BTG2 reduced the growth of BC3-p53KD cells in vitro relative to control cells (Figure S5C, D in Additional file 8). Reduced tumor growth was also observed in the mammary glands of engrafted mice (Fig. 4b) as well as in the lungs of mice after tail vein injection (Fig. 4c, d, e). Taken together, these results suggest that BTG2 functions downstream of p53 to negatively regulate growth of both primary and metastatic breast tumors.

### Expression of BTG2 correlates with breast cancer patient survival

**Fig. 5:**
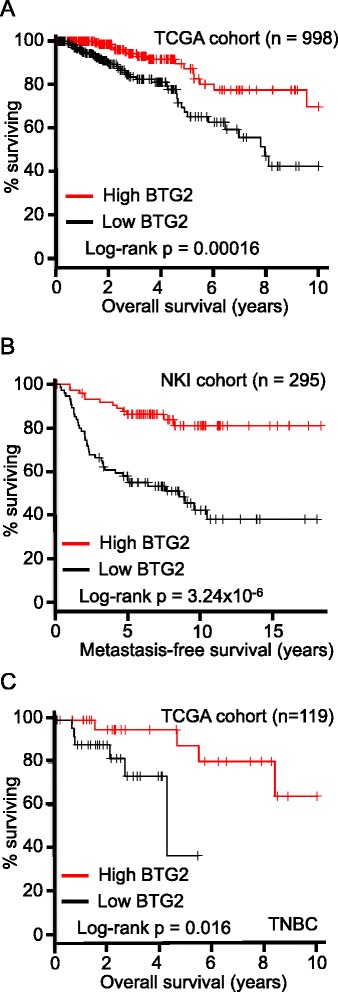
BTG2 is a clinically relevant modulator of tumor progression. Kaplan-Meier curves were generated to assess correlations between B cell translocation gene 2 (BTG2) expression and patient survival. Top and bottom 25 % of patients were used as cutoffs for grouping. **a** Low BTG2 expression correlates with decreased overall survival of breast cancer patients (all subtypes). **b** Low BTG2 expression correlates with decreased metastasis-free survival of breast cancer patients (all subtypes). **c** Low BTG2 expression correlates with decreased overall survival of triple-negative breast cancer (TNBC) patients. Number of patients and *p* values are indicated in each panel

To explore the prognostic relevance of BTG2 expression, survival analyses were performed. Low BTG2 expression correlated with decreased overall survival (Fig. 5a, Table S3 in Additional file 1) and metastasis-free survival (Fig. 5b) of breast cancer patients, indicating that BTG2 is a clinically relevant suppressor of breast tumor progression. In addition, low BTG2 expression correlated with decreased overall survival of patients with TNBC (Fig. 5c, Table S3 in Additional file 1). Thus, in addition to its predictive value in other subtypes of breast cancer, BTG2 expression may be a prognostic marker for TNBC.

## Discussion

Using a paired set of PDX models of TNBC differing only in p53 status that metastasize to similar organs as those observed in breast cancer patients, we demonstrated that p53 silencing enhanced tumor growth in both primary and metastatic sites. The BC3 lines used in this study were generated from a patient with metastatic breast cancer whose tumor was wild-type for p53. Thus, expression of wild-type p53 alone was insufficient to inhibit tumor metastasis in the patient and in PDX models derived from the patient’s tumor. However, p53 loss augmented metastatic potential by enhancing proliferation and reducing apoptosis and this, in turn, led to enhanced tumor growth. Thus, one contribution made by p53 loss to late-stage tumor progression was to promote tumor growth.

Previous studies conducted in vitro demonstrated that p53 inhibits pathways that promote tumor escape from the primary site, including ECM remodeling, connective tissue degradation, cell adhesion and EMT [3, 4]. Indeed, differential gene expression analysis revealed that these pathways were also deregulated upon p53 silencing in BC3 cells (Table S2 in Additional file 7). Despite these expression changes, p53 silencing in BC3 tumor cells did not significantly increase the shedding of CTCs into the bloodstream of tumor-bearing mice. However, based on the methods employed to detect CTCs, we cannot rule out the possibility that silencing of p53 increased CTC aggregation, thus improving their ability to seed secondary sites [41]. However, it seems more likely that CTC shedding was not affected by p53 status because the tumor line already possessed the pathway changes required for tumor escape.

p53 silencing resulted in the deregulation of many genes that likely contributed to enhanced tumor growth. It should be noted that in our study, gene expression changes resulting from p53 loss were identified by analyzing human breast tumor cells grown in the mammary fat pads of recipient mice. Mice were not exposed to DNA-damaging agents, although tumor cells are known to have intrinsic DNA damage. Troester et al. (2006) silenced p53 in established breast cancer cell lines and performed gene expression profiling on parental and p53-deficient cells both in the absence and presence of DNA damage [34]. Four of the differentially expressed genes identified in the absence of exogenous DNA damage in their study were also present on our list. These included *BTG2*, inositol polyphosphate-5-phosphatase (*INPP5D*), lysyl oxidase (*LOX*), and p21 protein (Cdc42/Rac)-activated kinase 6 (*PAK6*) (Table S1 in Additional file 6). In contrast to this in vitro analysis on nontransformed basal-like cell lines, our gene expression analyses were derived from PDX tumors that were engrafted in vivo. Thus, our analyses identified many alterations not observed in other studies. Importantly, BTG2 has been identified on both platforms, suggesting that it is a critical regulator of p53 function.

BTG2 is a direct transcriptional target of p53 [38–40] that regulates various cellular processes, including proliferation, differentiation, and apoptosis [38, 42, 43]. BTG2 expression is deregulated in various cancers, consistent with its role as a tumor suppressor protein. We observed reduced expression of BTG2 upon p53 silencing, and restoration of BTG2 expression in p53-deficient cells reduced proliferation in vitro and reduced breast tumor growth at both primary and secondary sites in vivo. In addition, reduced BTG2 expression correlated with reduced overall survival as well as metastasis-free survival of breast cancer patients, consistent with previous reports [42, 44–46]. Herein, we extended these findings by demonstrating that reduced BTG2 expression was associated with reduced overall survival in patients with TNBC. Thus, BTG2 functions as an important downstream effector of p53 to negatively regulate tumor progression and is a candidate prognostic biomarker for TNBC. Future experiments to examine the effect of BTG2 expression in additional PDX models will determine if BTG2 inhibits metastasis in multiple genetic backgrounds.

Although PDX models can largely recapitulate the heterogeneity of human tumors [47], it is generally acknowledged that they do not faithfully replicate human tumor - human stromal interactions. This is because human stroma is replaced by mouse stroma as a function of time after engraftment. In addition, PDX models generated in immune-compromised mice do not account for the important contributions made by immune cells to malignant progression. The immune system may limit or promote metastasis, and it is possible that WT or mutant p53 controls metastasis by signaling through immune components. Therefore, a thorough examination of the function of p53 in an immune-competent model is also warranted. Interestingly, Ding et al. (2010) reported that PDX models established from a TNBC of the basal subtype were more representative of the patient’s metastasis than her primary breast tumor [48]. Thus, data identifying these similarities between the metastasis and the xenograft validate the PDX model as a useful system for studying metastasis. Herein we report that human breast tumors were capable of completing all stages of the metastatic cascade in mice and metastatic lesions were observed in organs normally found in patients with metastatic breast cancer including lung, liver, bone, and brain. Thus, PDX models may provide a powerful system for delineating how heterogeneity contributes to metastatic progression and for identifying metastatic drivers that can then be functionally validated.

## Conclusions

A paired PDX set of tumors differing only in p53 status were used to study TNBC metastasis to lung, liver, bone, brain, and lymph nodes in vivo. These models were used to determine if p53 loss affects the metastatic potential of an already fully metastatic tumor. We demonstrated that p53 loss enhanced late-stage tumor progression by increasing proliferation and reducing apoptosis and this, in turn, led to enhanced tumor growth and cell shedding. Loss of BTG2, a p53 effector protein, contributed to the enhanced tumor growth and was associated with reduced overall survival in patients with TNBC. Thus, BTG2 is a candidate prognostic biomarker for TNBC.

## Additional files

Additional file 1: Table S3. Mini-signature of genes that interact with the p53 pathway and associated *p* values. (PDF 62 kb) Additional file 2: Figure S1. Silencing of p53 increased cell proliferation in vitro. BC3-p53WT and BC3-p53KD cells were plated on adherent cell culture plates. (A) Total photon flux from cultured cells was quantified in the presence of D-luciferin on an IVIS 100 immediately post-seeding. The specific bioluminescence of BC3-p53WT cells was brighter than that of BC3-p53KD cells; however, this difference did not adversely bias downstream analyses because BC3-p53KD cells grew and metastasized faster than BC3-p53WT, and any bias due to increased photon flux would be in favor of BC3-p53WT. Total photon flux was normalized to cell number. *p* = 0.002, *t* test. (B and C) BC3-p53WT and BC3-p53KD cells were plated on adherent cell culture plates, and growth was quantified by MTT assays (B) or cell proliferation assays (C). (D) Morphology of adherent BC3-p53WT and BC3-p53KD cells growing as two-dimensional cultures was captured by bright field microscopy. Magnified images are shown to the right of each image. All error bars represent standard deviation from the mean (SD). (PDF 977 kb) Additional file 3: Figure S2. p53 silencing does not increase number of tumor-initiating cells. (A and B) Limiting numbers of BC3-p53WT or BC3-p53KD were implanted to mouse mammary fat pads, and tumor presence and growth was assessed with BLI. Limiting dilution transplantation assays in immune-compromised mice demonstrated that as few as 10 BC3-p53WT and BC3-p53KD cells were capable of initiating tumors (A) that grew (exhibited increasing photon flux emission) over time (B). The presence of co-implanted fibroblasts did not alter the tumor-initiating capacity of limiting cell numbers. Error bars represent standard error of the mean (SEM). (C) One million BC3-p53WT and BC3-p53KD cells were implanted into mouse mammary fat pads, harvested at 0.5 cm diameter, and digested to single cells that were plated on low-attachment plates in the absence of serum as three-dimensional mammospheres. The number of established (primary) mammospheres is shown. *p* = 1.0. (D) Primary mammospheres from (C) were dissociated to single cells and replated on low attachment plates in the absence of serum. The number of established (secondary) mammospheres is shown. *p* = 0.06. Error bars represent standard deviation from the mean (SD). (E) One million BC3-p53WT or BC3-p53KD cells were implanted into mouse mammary fat pads and harvested when they reached 0.5 cm diameter. Tumors were digested to single cells and stained with antibodies for flow cytometry to assess expression of CD24 and CD44. The percentage of cells from BC3-p53WT and BC3-p53KD mammary tumors that exhibit high levels of CD44 and low levels of CD24 is shown. *p* = 0.06. Error bars represent standard deviation from the mean (SD) (C-E). Wilcoxon rank sum tests were used for statistical analyses. (PDF 134 kb) Additional file 4: Figure S3. p53 silencing increases growth at metastatic sites. BC3-p53WT or BC3-p53KD cells were injected to the left cardiac ventricle (A, *p* = 0.22 at 4 weeks; *p* = 0.01 at 6 weeks)) or tail vein (B, *p* = 0.03 at 13 weeks), and mice were subjected to BLI in vivo at the indicated time points. Total photon flux was assessed on an IVIS SPECTRUM and quantified. Each data point represents one mouse. All error bars represent standard error of the mean (SEM). Wilcoxon rank sum tests were used for statistical analyses. (PDF 73 kb) Additional file 5: Figure S4. CTC number correlates with mammary tumor size. Mouse mammary fat pads were engrafted with BC3-p53WT or BC3-p53KD. Whole blood was extracted from mice in a terminal blood draw by cardiac puncture. Red blood cells were lysed, and circulating tumor cells (CTCs) were assessed by flow cytometry for mCherry-positive cells. BLI was performed on mammary tumors and lungs at necropsy. (A and B) CTCs were quantified by flow cytometry and normalized to total photon flux (A) or volume (B) of each corresponding mammary tumor at indicated time points. Error bars represent standard error of the mean (SEM). (C) CTC number was plotted versus photon flux of the mammary tumor within each mouse. Pearson correlation = 0.82, *p* <0.001. (D) CTC number was plotted versus tumor volume within each mouse. Pearson correlation = 0.61, *p* = 0.001. (E) CTC number was plotted versus photon flux in the lung. Pearson correlation = 0.79, *p* <0.001. (F) CTC number was plotted versus time of lymph node metastasis detection. Pearson correlation = 0.36, *p* = 0.23. Each data point represents one mouse. (PDF 2462 kb) Additional file 6: Table S1. RNA-Seq signature of genes that are deregulated upon p53 loss in BC3 mammary tumors. (PDF 123 kb) Additional file 7: Table S2. Pathway analysis of RNA-Seq expression signature consisting of genes deregulated upon p53 knockdown. (PDF 63 kb) Additional file 8: Figure S5. Ectopic expression of BTG2 in BC3-p53KD cells reduces cell proliferation in vitro. BC3-p53KD cells were stably transduced with plasmids encoding BTG2 or GFP (control). mRNA (A) and protein (B) levels of BTG2 were determined by qRT-PCR and Western blotting. Cell proliferation was assessed in vitro using cell proliferation assays (C) or MTT assays (D and E). Error bars represent standard deviation from the mean (SD). (PDF 1964 kb)

## Abbreviations

ANOVA: Analysis of variance
APC: Allophycocyanin
BLI: Bioluminescence imaging
BSA: Bovine serum albumin
BTG2: B cell translocation gene 2
CBR-Luc: Click beetle red luciferase
CC3: Cleaved caspase 3
CK18: Cytokeratin 18
CTC: Circulating tumor cell
DMEM: Dulbecco’s modified Eagle’s medium
DTT: Dithiothreitol
ECL: Enhanced chemiluminescence
ECM: Extracellular matrix
EDTA: Ethylenediaminetetraacetic acid
EMT: Epithelial mesenchymal transition
FBS: Fetal bovine serum
FITC: Fluorescein isothiocyanate
GFP: Green fluorescent protein
HER2-E: HER2 enriched
IACUC: Institutional Animal Care and Use Committee
IHC: Immunohistochemistry
INPP5D: Inositol polyphosphate-5-phosphatase 5D
KD: Knockdown
LOX: Lysyl oxidase
MOI: Multiplicity of infection
MTT: 3-(4,5-dimethylthiazol-2-yl)-2,5-diphenyltetrazolium bromide
NOD/SCID: Nonobese diabetic/severe combined immunodeficiency
PAK6: P21 protein (Cdc42/Rac)-activated kinase 6
PBS: phosphate-buffered saline
PDX: Patient-derived xenograft
pHH3: Phospho-histone H3
PMSF: Phenylmethanesulfonyl fluoride
qPCR: Quantitative polymerase chain reaction
RBC: Red blood cell
RIN: RNA integrity number
RNA-Seq: Ribonucleic acid-sequencing
RT: Reverse transcription
RT-PCR: Real-time polymerase chain reaction
SD: Standard deviation
SEM: Standard error of the mean
shRNA: Short hairpin RNA
TCGA: The Cancer Genome Atlas
TNBC: Triple-negative breast cancer
TP53: Gene encoding p53
WT: Wild-type
WU-BC3: Washington University- Breast Cancer line 3
