# Supplementary material for: p53 deficiency linked to B cell translocation gene 2 (BTG2) loss enhances metastatic potential by promoting tumor growth in primary and metastatic sites in patient-derived xenograft (PDX) models of triple-negative breast cancer
Source: Breast Cancer Res. 2016 Jan 27;18:13. doi: 10.1186/s13058-016-0673-9 (PMC4728775; doi:10.1186/s13058-016-0673-9)
Supplement: Additional file 1: Table S3. — Mini-signature of genes that interact with the p53 pathway and associated p values. (PDF 62 kb) [file 13058_2016_673_MOESM1_ESM.pdf]

Table S3. Mini-signature of genes that interact with the p53 pathway and associated p-values.

| **p53 associated genes** | **TCGA cohort (all breast cancer) Overall Survival (n=1022)** | **TCGA cohort (TNBC) overall survival (n=119)** | **San Diego cohort (metastasis free survival) (n=286)** | **NKI cohort (metastasis free survival) (n=295)** | **Oxford cohort (metastasis free survival) (n=210)** |
|---|---|---|---|---|---|
| GDF15 | 0.618704 | 0.872751 | 0.512639 | NA | NA |
| SPATA18 | 0.931971 | 0.909813 | NA | NA | NA |
| FAM27A | 0.795673 | 0.738823 | NA | NA | NA |
| BTG2 | 0.000866 | 0.125703 | 0.181338 | 0.000003 | 0.000168 |
| CA9 | 0.429666 | 0.538078 | 0.318494 | 0.001134 | 0.000787 |
| TP53 | 0.406990 | 0.717982 | 0.892629 | 0.902928 | 0.748604 |
| EDA2R | 0.003220 | 0.493069 | 0.517135 | NA | NA |
| NME1 | 0.394788 | 0.386684 | 0.043214 | 0.124441 | 0.020291 |
| NME2 | 0.017816 | 0.401633 | NA | 0.407861 | 0.164076 |
| TNFRSF1B | 0.030622 | 0.737269 | 0.014245 | 0.967189 | 0.655479 |
| ACTA2 | 0.128326 | 0.584963 | 0.010685 | 0.759370 | 0.633141 |
| PRAP1 | 0.179881 | 0.469873 | NA | NA | NA |
| DSG3 | 0.015148 | 0.281834 | 0.661268 | 0.404740 | 0.734274 |
| EDIL3 | 0.019433 | 0.253810 | 0.138877 | 0.491648 | 0.323478 |
| BHLHE41 | 0.192112 | 0.463182 | 0.868209 | NA | NA |
| MMP2 | 0.744432 | 0.927997 | 0.779695 | 0.607070 | 0.558907 |
| PCDH7 | 0.527964 | 0.473452 | 0.718918 | 0.506622 | 0.990493 |
| THBS1 | 0.027219 | 0.234703 | 0.260597 | 0.413557 | 0.092741 |
| PTX3 | 0.237603 | 0.747768 | 0.089691 | 0.659664 | 0.746536 |
| SEMA3A | 0.472948 | 0.127119 | 0.057343 | 0.955063 | 0.500024 |
| LUM | 0.577808 | 0.320572 | 0.436260 | 0.060627 | 0.726615 |
| EPHA4 | 0.527416 | 0.561032 | 0.682572 | 0.121261 | 0.144562 |
| KRT14 | 0.002357 | 0.723528 | 0.387805 | 0.700514 | 0.561103 |
| TNFSF14 | 0.216639 | 0.738366 | 0.820042 | 0.708935 | 0.602292 |
| NLRP3 | 0.276127 | 0.994360 | 0.876382 | NA | NA |
| IGFBP7 | 0.098384 | 0.903900 | 0.071742 | 0.828768 | 0.605262 |
| SP7 | 0.071958 | 0.034029 | NA | NA | 0.451514 |
| MMP16 | 0.275221 | 0.149805 | 0.475863 | 0.439643 | 0.277535 |
| ZACN | 0.113341 | 0.285311 | NA | NA | NA |
| RUNX2 | 0.190225 | 0.257235 | 0.362197 | 0.901616 | 0.616612 |
| PAK6 | 0.046595 | 0.367752 | 0.684316 | 0.669276 | 0.328800 |

*Red shading indicates significant p-value
