# Supplementary figures and images for: p53 deficiency linked to B cell translocation gene 2 (BTG2) loss enhances metastatic potential by promoting tumor growth in primary and metastatic sites in patient-derived xenograft (PDX) models of triple-negative breast cancer
Source: Breast Cancer Res. 2016 Jan 27;18:13. doi: 10.1186/s13058-016-0673-9 (PMC4728775; doi:10.1186/s13058-016-0673-9)

A

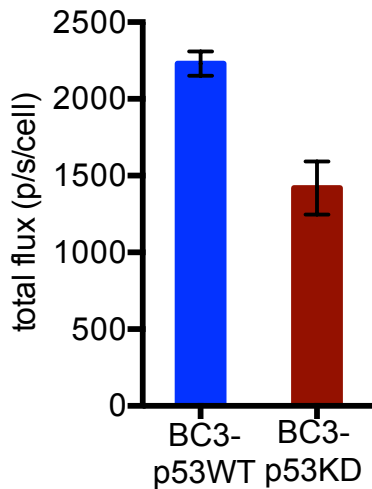

C

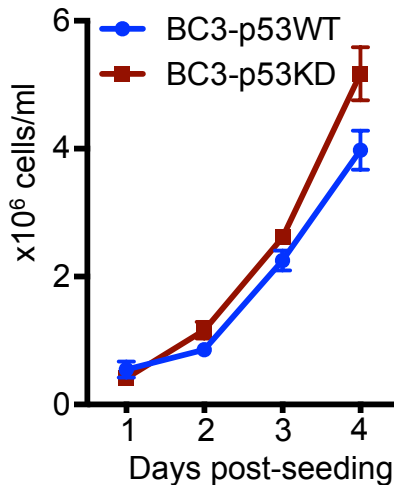

B

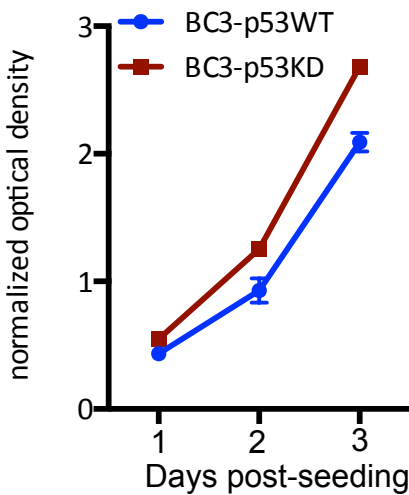

D

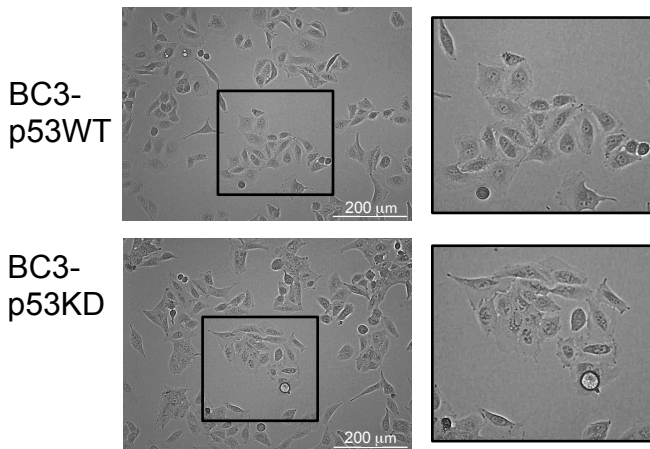

Supplement: Additional file 2: Figure S1. — Silencing of p53 increased cell proliferation in vitro. BC3-p53WT and BC3-p53KD cells were plated on adherent cell culture plates. (A) Total photon flux from cultured cells was quantified in the presence of D-luciferin on an IVIS 100 immediately post-seeding. The specific bioluminescence of BC3-p53WT cells was brighter than that of BC3-p53KD cells; however, this difference did not adversely bias downstream analyses because BC3-p53KD cells grew and metastasized faster than BC3-p53WT, and any bias due to increased photon flux would be in favor of BC3-p53WT. Total photon flux was normalized to cell number. p = 0.002, t test. (B and C) BC3-p53WT and BC3-p53KD cells were plated on adherent cell culture plates, and growth was quantified by MTT assays (B) or cell proliferation assays (C). (D) Morphology of adherent BC3-p53WT and BC3-p53KD cells growing as two-dimensional cultures was captured by bright field microscopy. Magnified images are shown to the right of each image. All error bars represent standard deviation from the mean (SD). (PDF 977 kb) [file 13058_2016_673_MOESM2_ESM.pdf]

A

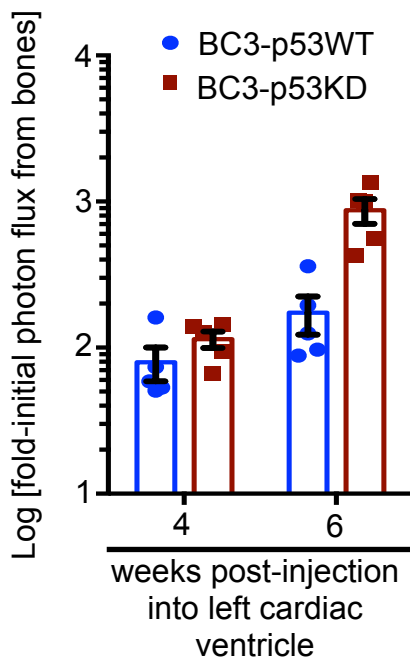

B

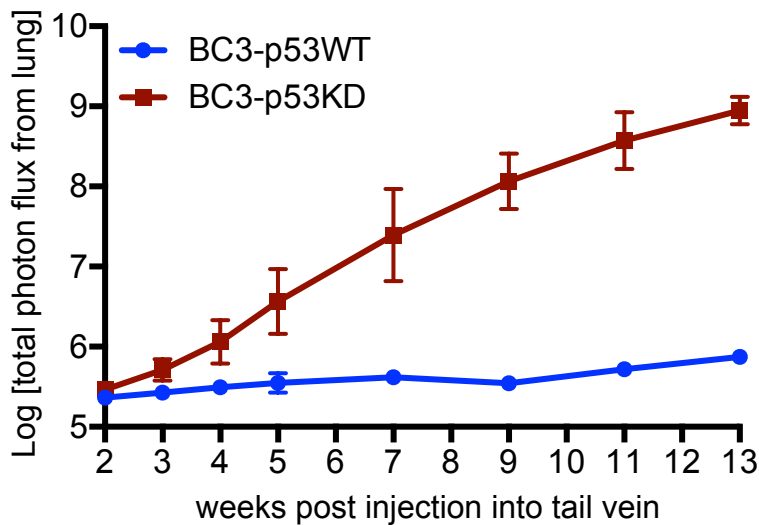

Supplement: Additional file 4: Figure S3. — p53 silencing increases growth at metastatic sites. BC3-p53WT or BC3-p53KD cells were injected to the left cardiac ventricle (A, p = 0.22 at 4 weeks; p = 0.01 at 6 weeks)) or tail vein (B, p = 0.03 at 13 weeks), and mice were subjected to BLI in vivo at the indicated time points. Total photon flux was assessed on an IVIS SPECTRUM and quantified. Each data point represents one mouse. All error bars represent standard error of the mean (SEM). Wilcoxon rank sum tests were used for statistical analyses. (PDF 73 kb) [file 13058_2016_673_MOESM4_ESM.pdf]

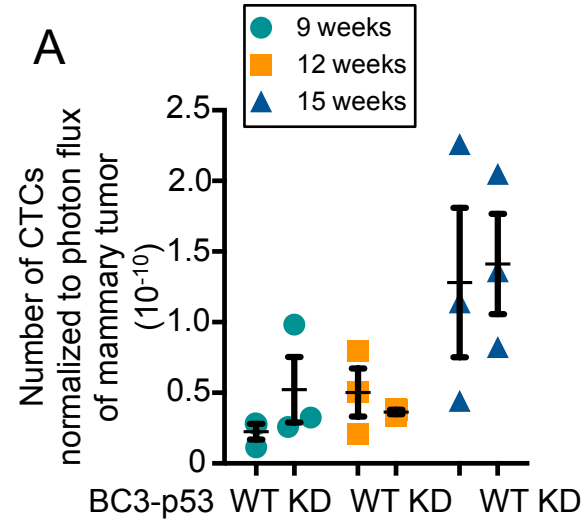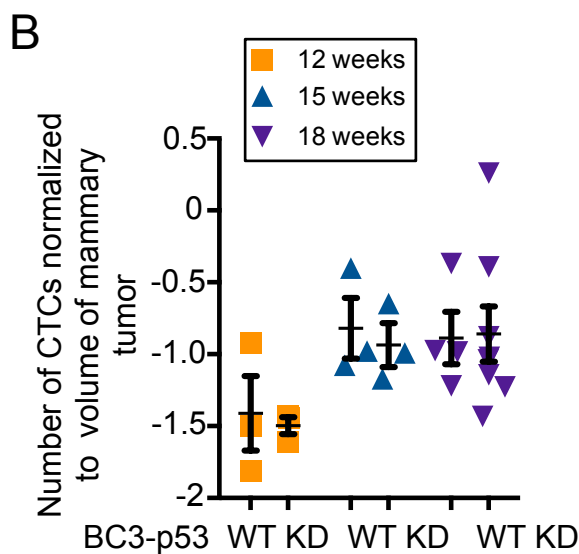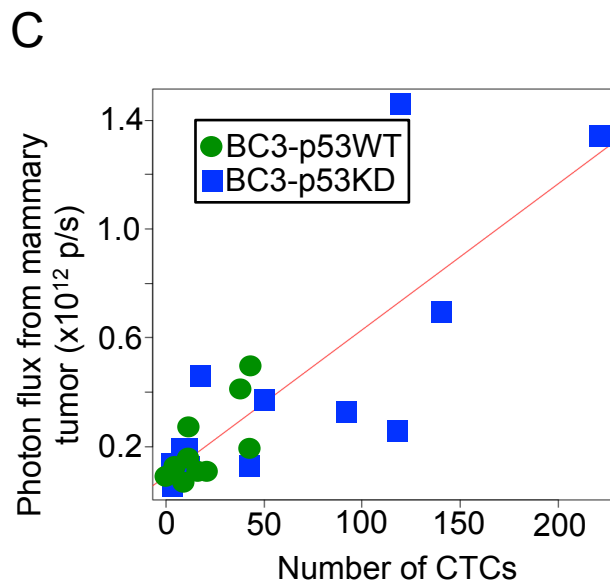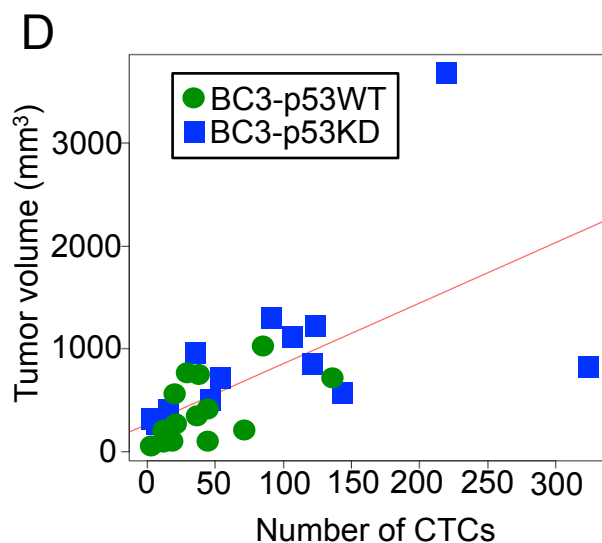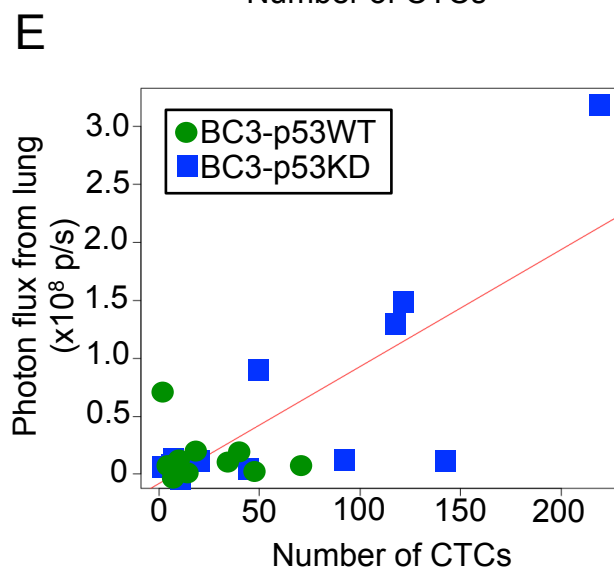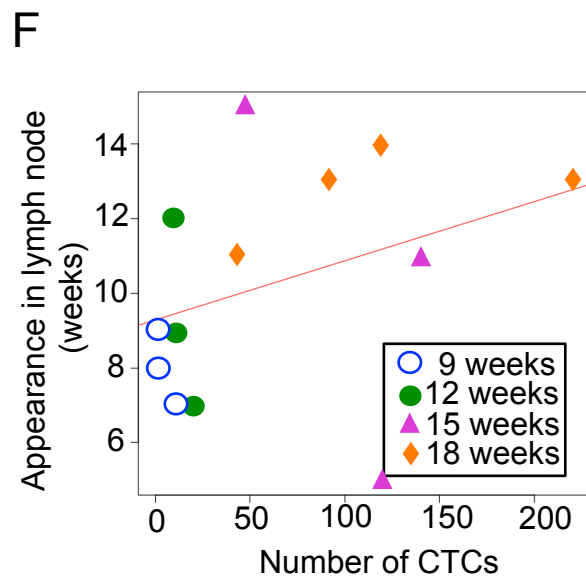

Supplement: Additional file 5: Figure S4. — CTC number correlates with mammary tumor size. Mouse mammary fat pads were engrafted with BC3-p53WT or BC3-p53KD. Whole blood was extracted from mice in a terminal blood draw by cardiac puncture. Red blood cells were lysed, and circulating tumor cells (CTCs) were assessed by flow cytometry for mCherry-positive cells. BLI was performed on mammary tumors and lungs at necropsy. (A and B) CTCs were quantified by flow cytometry and normalized to total photon flux (A) or volume (B) of each corresponding mammary tumor at indicated time points. Error bars represent standard error of the mean (SEM). (C) CTC number was plotted versus photon flux of the mammary tumor within each mouse. Pearson correlation = 0.82, p <0.001. (D) CTC number was plotted versus tumor volume within each mouse. Pearson correlation = 0.61, p = 0.001. (E) CTC number was plotted versus photon flux in the lung. Pearson correlation = 0.79, p <0.001. (F) CTC number was plotted versus time of lymph node metastasis detection. Pearson correlation = 0.36, p = 0.23. Each data point represents one mouse. (PDF 2462 kb) [file 13058_2016_673_MOESM5_ESM.pdf]

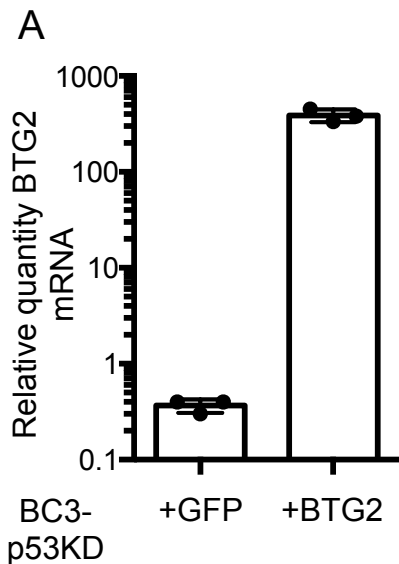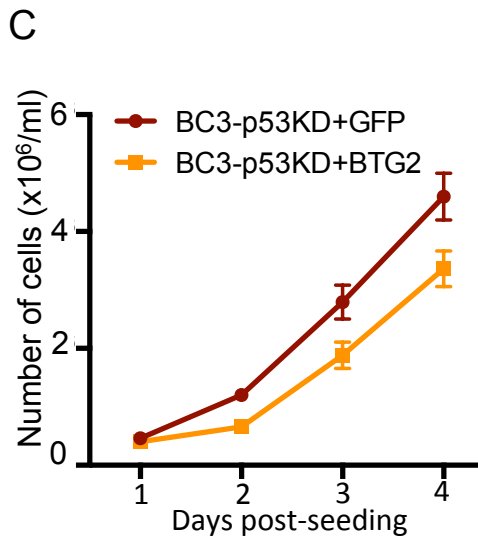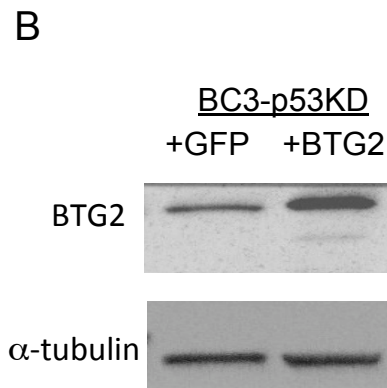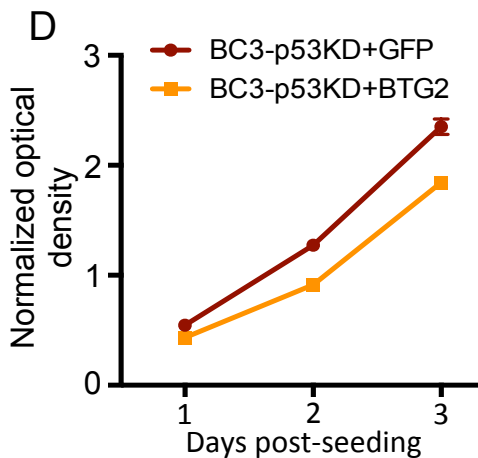

Supplement: Additional file 8: Figure S5. — Ectopic expression of BTG2 in BC3-p53KD cells reduces cell proliferation in vitro. BC3-p53KD cells were stably transduced with plasmids encoding BTG2 or GFP (control). mRNA (A) and protein (B) levels of BTG2 were determined by qRT-PCR and Western blotting. Cell proliferation was assessed in vitro using cell proliferation assays (C) or MTT assays (D and E). Error bars represent standard deviation from the mean (SD). (PDF 1964 kb) [file 13058_2016_673_MOESM8_ESM.pdf]
