# Supplementary material for: p53 deficiency linked to B cell translocation gene 2 (BTG2) loss enhances metastatic potential by promoting tumor growth in primary and metastatic sites in patient-derived xenograft (PDX) models of triple-negative breast cancer
Source: Breast Cancer Res. 2016 Jan 27;18:13. doi: 10.1186/s13058-016-0673-9 (PMC4728775; doi:10.1186/s13058-016-0673-9)

A

	Number of cells implanted	Number of BC3-p53WT tumors developed	Number of BC3-p53KD tumors developed
Number of tumor cells injected co-implanted with fibroblasts	10 cells	10/10	10/10
	100 cells	8/8	10/10
	1,000 cells	4/4	4/4
	10,000 cells	4/4	4/4
Number of tumor cells implanted in absence of fibroblasts	10 cells	6/6	5/6
	100 cells	4/4	4/4

B

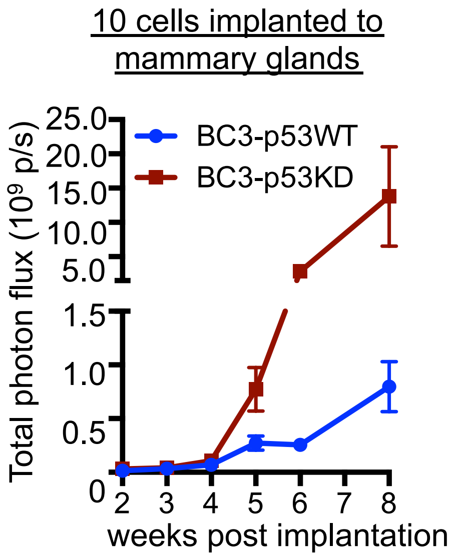

C

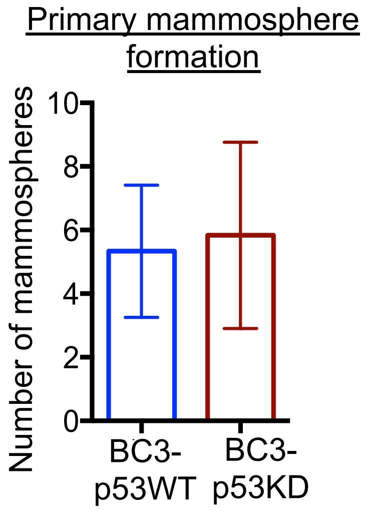

D

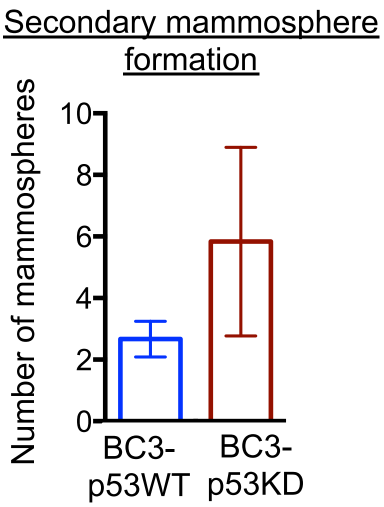

E

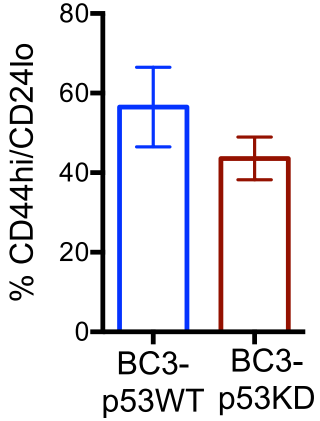

Supplement: Additional file 3: Figure S2. — p53 silencing does not increase number of tumor-initiating cells. (A and B) Limiting numbers of BC3-p53WT or BC3-p53KD were implanted to mouse mammary fat pads, and tumor presence and growth was assessed with BLI. Limiting dilution transplantation assays in immune-compromised mice demonstrated that as few as 10 BC3-p53WT and BC3-p53KD cells were capable of initiating tumors (A) that grew (exhibited increasing photon flux emission) over time (B). The presence of co-implanted fibroblasts did not alter the tumor-initiating capacity of limiting cell numbers. Error bars represent standard error of the mean (SEM). (C) One million BC3-p53WT and BC3-p53KD cells were implanted into mouse mammary fat pads, harvested at 0.5 cm diameter, and digested to single cells that were plated on low-attachment plates in the absence of serum as three-dimensional mammospheres. The number of established (primary) mammospheres is shown. p = 1.0. (D) Primary mammospheres from (C) were dissociated to single cells and replated on low attachment plates in the absence of serum. The number of established (secondary) mammospheres is shown. p = 0.06. Error bars represent standard deviation from the mean (SD). (E) One million BC3-p53WT or BC3-p53KD cells were implanted into mouse mammary fat pads and harvested when they reached 0.5 cm diameter. Tumors were digested to single cells and stained with antibodies for flow cytometry to assess expression of CD24 and CD44. The percentage of cells from BC3-p53WT and BC3-p53KD mammary tumors that exhibit high levels of CD44 and low levels of CD24 is shown. p = 0.06. Error bars represent standard deviation from the mean (SD) (C-E). Wilcoxon rank sum tests were used for statistical analyses. (PDF 134 kb) [file 13058_2016_673_MOESM3_ESM.pdf]
