# Supplementary material for: p53 deficiency linked to B cell translocation gene 2 (BTG2) loss enhances metastatic potential by promoting tumor growth in primary and metastatic sites in patient-derived xenograft (PDX) models of triple-negative breast cancer
Source: Breast Cancer Res. 2016 Jan 27;18:13. doi: 10.1186/s13058-016-0673-9 (PMC4728775; doi:10.1186/s13058-016-0673-9)
Supplement: Additional file 6: Table S1. — RNA-Seq signature of genes that are deregulated upon p53 loss in BC3 mammary tumors. (PDF 123 kb) [file 13058_2016_673_MOESM6_ESM.pdf]

Table S1. RNA-Seq signature of genes that are de-regulated upon p53 loss in BC3 mammary tumors

BC3-p53WT vs. BC3-p53KD mammary tumor gene expression signature				
Gene Symbol	log2 Fold Change	actual fold change	pvalue (Wald test p-value)	expression increased or decreased when p53 is silenced
TP53	3.4	10.9	7.84E-30	decreased
SGCZ	3.3	9.9	6.36E-07	decreased
NARR	3.2	9.5	6.33E-07	decreased
INPP5D	3.0	7.7	3.72E-13	decreased
EDA2R	2.8	7.2	1.23E-09	decreased
MIR3655	2.7	6.4	1.73E-05	decreased
RAD51L3-RFFL	2.5	5.5	2.29E-04	decreased
LINC01021	2.3	5.0	7.07E-05	decreased
SLC22A11	2.1	4.3	4.63E-05	decreased
CA9	2.1	4.2	8.08E-06	decreased
ABCC6P1	2.0	4.0	3.08E-03	decreased
TREML3P	2.0	4.0	1.05E-04	decreased
PPP1R14D	2.0	4.0	4.05E-04	decreased
GABRG1	2.0	3.9	3.80E-03	decreased
ADAM21	1.9	3.8	2.55E-03	decreased
C19orf83	1.9	3.7	1.00E-04	decreased
ANP32D	1.9	3.7	5.00E-03	decreased
SPATA18	1.8	3.6	4.52E-04	decreased
GDF15	1.8	3.5	2.30E-08	decreased
CCDC178	1.8	3.4	8.87E-03	decreased
C18orf56	1.8	3.4	1.57E-04	decreased
VWCE	1.8	3.4	4.55E-03	decreased
CECR7	1.8	3.4	1.83E-04	decreased
TMEM82	1.7	3.4	2.32E-03	decreased
SLC51B	1.7	3.3	3.44E-04	decreased
APOC1	1.7	3.2	7.64E-04	decreased
PSG3	1.7	3.2	9.60E-05	decreased
ARX	1.7	3.1	6.23E-03	decreased
ZNF141	1.6	3.1	1.15E-02	decreased
HABP2	1.6	3.1	1.08E-02	decreased
CD163	1.6	3.1	1.70E-02	decreased
CREB3L1	1.6	3.0	8.32E-03	decreased
ADORA1	1.6	3.0	3.38E-04	decreased

VSIG10L	1.6	3.0	5.63E-04	decreased
FAM27A	1.6	3.0	2.87E-03	decreased
NDUFA4L2	1.5	2.9	2.54E-03	decreased
RPS14P3	1.5	2.9	6.64E-03	decreased
WNT10B	1.5	2.9	2.39E-04	decreased
EIF4EBP3	1.5	2.9	6.72E-03	decreased
TNFRSF1B	1.5	2.9	2.33E-03	decreased
BTG2	1.5	2.9	1.92E-05	decreased
NME1-NME2	1.5	2.8	2.51E-02	decreased
ACTA2	1.5	2.8	3.00E-04	decreased
CACNA2D4	1.5	2.8	2.48E-03	decreased
PRAP1	1.5	2.8	5.81E-03	decreased
ASGR2	1.5	2.8	7.71E-03	decreased
PALD1	1.5	2.8	2.41E-03	decreased
BATF2	1.5	2.8	8.63E-03	decreased
LINC00261	1.5	2.7	1.59E-02	decreased
LOC102467081	1.5	2.7	2.73E-02	decreased
RPL22L1	1.5	2.7	1.00E-02	decreased
DSG3	3.2	9.0	3.63E-10	increased
ISPD	2.7	6.7	3.41E-05	increased
DDX11L9	2.7	6.5	7.16E-05	increased
ITGB8	2.6	5.9	2.53E-06	increased
EDIL3	2.6	5.9	3.05E-05	increased
PKP1	2.6	5.9	1.07E-04	increased
HOXC11	2.4	5.1	1.88E-04	increased
KLF8	2.3	5.1	2.26E-05	increased
BHLHE41	2.3	5.0	1.96E-04	increased
EPB41L3	2.3	4.9	2.15E-04	increased
CLIC6	2.2	4.5	1.24E-03	increased
MMP2	2.1	4.4	1.08E-04	increased
NTS	2.1	4.4	1.59E-03	increased
MB21D1	2.1	4.4	1.72E-03	increased
LRRTM3	2.1	4.2	2.03E-03	increased
PRR16	2.1	4.2	1.99E-03	increased
BLACAT1	2.0	4.1	1.11E-04	increased
NPM2	2.0	4.1	2.41E-03	increased
PCDH1	2.0	4.0	5.26E-05	increased
C10orf10	2.0	4.0	1.77E-04	increased
MFAP5	2.0	4.0	3.54E-04	increased
PTX3	2.0	3.9	3.92E-03	increased
PCBP3	1.9	3.8	2.04E-03	increased

C15orf56	1.9	3.8	4.11E-03	increased
TM4SF18	1.9	3.8	4.79E-03	increased
KRT6A	1.9	3.8	1.74E-03	increased
SERPINA3	1.9	3.7	4.88E-04	increased
ACTL8	1.9	3.7	1.86E-03	increased
CCL28	1.9	3.6	6.01E-04	increased
MAP3K7CL	1.8	3.6	1.74E-03	increased
FAP	1.8	3.6	3.35E-03	increased
ARID5B	1.8	3.6	1.83E-03	increased
SMIM10	1.8	3.5	7.20E-03	increased
SEMA3A	1.8	3.5	4.48E-04	increased
LOX	1.8	3.5	2.60E-06	increased
STL	1.8	3.5	5.99E-03	increased
LINGO2	1.8	3.4	5.87E-03	increased
RPS6KA2	1.8	3.4	1.38E-03	increased
GAS6-AS2	1.8	3.4	4.20E-03	increased
ACTR3C	1.8	3.4	8.44E-03	increased
TSPY26P	1.8	3.4	7.32E-03	increased
LUM	1.7	3.4	1.24E-03	increased
ZNF608	1.7	3.3	1.99E-03	increased
GJA1	1.7	3.3	3.88E-03	increased
MMP19	1.7	3.3	8.96E-03	increased
EPHA4	1.7	3.3	2.35E-03	increased
SPOCK3	1.7	3.3	9.60E-03	increased
EPHX4	1.7	3.3	3.01E-03	increased
CLIC5	1.7	3.3	2.78E-03	increased
GPR110	1.7	3.2	8.80E-03	increased
HOTAIR	1.7	3.2	1.27E-02	increased
MAOB	1.7	3.2	3.53E-03	increased
MMP7	1.7	3.2	1.33E-03	increased
PRKACB	1.7	3.2	6.96E-05	increased
RIMS1	1.7	3.2	1.34E-02	increased
TMPRSS4	1.7	3.2	1.20E-03	increased
KRT14	1.7	3.2	9.22E-03	increased
TNFSF14	1.7	3.2	1.11E-02	increased
NLRP3	1.7	3.2	9.49E-03	increased
TMEFF2	1.7	3.1	1.47E-02	increased
EDAR	1.6	3.1	1.86E-03	increased
CCDC85A	1.6	3.1	2.34E-04	increased
DUSP27	1.6	3.1	1.50E-02	increased
LOC340113	1.6	3.1	1.56E-02	increased

LOC100379224	1.6	3.1	4.11E-03	increased
FHOD3	1.6	3.1	1.45E-03	increased
IGFBP7	1.6	3.1	4.47E-03	increased
POU4F3	1.6	3.0	1.83E-02	increased
BMP6	1.6	3.0	7.39E-04	increased
SP7	1.6	3.0	1.59E-02	increased
RFTN2	1.6	3.0	1.96E-02	increased
KIAA1644	1.6	3.0	1.81E-02	increased
PCDH7	1.6	3.0	6.14E-03	increased
PELI1	1.6	3.0	2.62E-04	increased
GRIK1	1.6	3.0	1.88E-02	increased
MMP16	1.6	3.0	1.92E-02	increased
MOV10L1	1.6	3.0	1.74E-02	increased
HMCN1	1.6	3.0	1.92E-02	increased
KCNA7	1.6	2.9	1.11E-02	increased
MAML2	1.6	2.9	3.37E-04	increased
ZNF57	1.5	2.9	1.19E-02	increased
SOX8	1.5	2.9	1.79E-02	increased
LINC00163	1.5	2.9	1.25E-02	increased
THBS1	1.5	2.9	4.00E-03	increased
LOC100129027	1.5	2.9	1.71E-02	increased
MUC16	1.5	2.9	1.64E-02	increased
GRIN3B	1.5	2.8	2.77E-03	increased
GHRLOS	1.5	2.8	2.55E-02	increased
ROBO1	1.5	2.8	1.99E-02	increased
C4orf26	1.5	2.8	1.74E-02	increased
ZACN	1.5	2.8	2.65E-02	increased
FGB	1.5	2.8	1.66E-02	increased
RNF165	1.5	2.8	2.89E-02	increased
SLC16A7	1.5	2.8	5.92E-03	increased
CCL24	1.5	2.8	1.59E-02	increased
DNAH10	1.5	2.8	1.53E-02	increased
RUNX2	1.5	2.8	2.79E-02	increased
SLITRK6	1.5	2.8	2.80E-02	increased
IRAK3	1.5	2.8	3.02E-02	increased
PITPNM3	1.5	2.8	9.39E-03	increased
RYR2	1.5	2.8	2.97E-02	increased
B3GNT6	1.5	2.8	1.61E-02	increased
PAK6	1.5	2.8	2.08E-03	increased
PLCL1	1.5	2.8	2.46E-02	increased
JAM2	1.5	2.8	2.65E-02	increased

SH3TC2	1.5	2.8	9.77E-03	increased
UBOX5-AS1	1.5	2.7	2.94E-02	increased
SOX21-AS1	1.5	2.7	3.17E-02	increased
HR	1.5	2.7	9.98E-03	increased
