# Supplementary material for: p53 deficiency linked to B cell translocation gene 2 (BTG2) loss enhances metastatic potential by promoting tumor growth in primary and metastatic sites in patient-derived xenograft (PDX) models of triple-negative breast cancer
Source: Breast Cancer Res. 2016 Jan 27;18:13. doi: 10.1186/s13058-016-0673-9 (PMC4728775; doi:10.1186/s13058-016-0673-9)
Supplement: Additional file 7: Table S2. — Pathway analysis of RNA-Seq expression signature consisting of genes deregulated upon p53 knockdown. (PDF 63 kb) [file 13058_2016_673_MOESM7_ESM.pdf]

Table S2. Pathway analysis of RNA-Seq expression signature consisting of genes de-regulated upon p53 knockdown

#	Networks	Total	p-value	FDR	# genes in data	Genes Deregulated
1	Proteolysis_ECM remodeling	85	9.426 E-06	5.314 E-04	7	MMP-7, MMP-19, MMP-16, SPOCK3, SERPINA3, MMP-2, Lumican
2	Proteolysis_Connective tissue degradation	119	9.489 E-06	5.314 E-04	8	MMP-7, MMP-19, MMP-16, SPOCK3, SERPINA3, MMP-2, Lumican, HABP2
3	Cell adhesion_Cell-matrix interactions	211	9.669 E-05	3.610 E-03	9	MMP-7, MMP-19, GJA1, MMP-16, MMP-2, THBS1, Lumican, HABP2, SGCZ
4	Development_Neurogenesis_Synaptogenesis	180	1.059 E-03	2.965 E-02	5	RIMS1, GRIK1, THBS1, WNT10B, ACTA2
5	Apoptosis_Anti-apoptosis mediated by external signals via NF-κB	111	3.040 E-03	6.413 E-02	5	PRKACB, RPS6KA2, p53, TNFRSF1B, ADORA1
6	Development_EMT_Regulation of epithelial-to-mesenchymal transition	225	3.774 E-03	6.413 E-02	6	KRT14, LOX, RPS6KA2, MMP-2, WNT10B, ACTA2
7	Development_Neurogenesis_Axonal guidance	230	4.259 E-03	6.413 E-02	7	EPHA4, CCL28, ROBO1, SEMA3A, PRKACB, ACTA2
8	Cell adhesion_Attractive and repulsive receptors	175	4.581 E-03	6.413 E-02	6	EPHA4, ROBO1, SEMA3A, PRKACB, ACTA2
9	Cell adhesion_Cadherins	180	5.252 E-03	6.512 E-02	6	PKP1, DSG3, PCDH7, ACTA2, WNT10B
10	Cytoskeleton_Intermediate filaments	81	5.814 E-03	6.512 E-02	4	PKP1, KRT14,KRT6A, ACTA2

green text indicates up-regulation in response to p53 knockdown

red text indicates down-regulation in response to p53 knockdown
